# Are Long-Chain Polyunsaturated Fatty Acids the Link between the Immune System and the Microbiome towards Modulating Cancer?

**DOI:** 10.3390/medicines5030102

**Published:** 2018-09-10

**Authors:** Leodevico L. Ilag

**Affiliations:** Xerion Limited, Brighton, 3186 Victoria, Australia; vilag@xerion.com.au; Tel.: +61-395-538-868

**Keywords:** inflammation, polyunsaturated fatty acids, omega-3 fatty acids, omega-6 fatty acids, microbiome, immune checkpoint inhibitors, immunotherapy, cancer, gut health, dysbiosis

## Abstract

Three recent studies revealed synergy between immune-checkpoint inhibitors and the microbiome as a new approach in the treatment of cancer. Incidentally, there has been significant progress in understanding the role of polyunsaturated fatty acids (PUFAs) in modulating cancer and the immune system, as well as in regulating the microbiome. Inflammation seems to be the common denominator among these seemingly unrelated biological entities—immune system, the microbiome, and long-chain polyunsaturated fatty acids (LC-PUFAs). This commentary presents a hypothesis proposing the existence of an optimal level of LC-PUFAs that nurtures the suitable gut microbiota preventing dysbiosis. This synergy between optimal LC-PUFAs and gut microbiota helps the immune system overcome the immunosuppressive tumour microenvironment including enhancing the efficacy of immune checkpoint inhibitors. A model on how LC-PUFAs (such as omega(n)-3 and n-6 fatty acids) forms a synergistic triad with the immune system and the microbiome in regulating inflammation to maintain homeostasis is presented. The principles underlying the hypothesis provide a basis in managing and even preventing cancer and other chronic diseases associated with inflammation.

## 1. Introduction

The past decade has witnessed exciting developments in the medical field with the advancement of immunotherapy in managing cancer and the microbiome as a potential therapeutic modality. One of the challenges in immunotherapy is the cunning ability of cancer cells to evade the patients’ immune system. This has been attributed to the tumour cells’ ability to exploit the inflammatory microenvironment around the tumour, which promotes immunosuppression or the inability of the host’s immune surveillance system to recognise the tumour as foreign. A breakthrough came with the understanding of a tumour’s escape mechanism using the programmed death (PD-1) pathway. In this pathway, programmed death ligand 1 (PD-L1), which is often overexpressed on tumour cells, negatively regulates T-cell mediated response upon binding to the immune cells’ PD-1 receptor, allowing tumours to evade the T-cell immune response. This mechanism of evasion has been termed the immune checkpoint. Development of inhibitors of immune checkpoint pathway has led to successful therapeutic antibodies against PD-1 (nivolumumab and pembrolizumab) that has shown a therapeutic benefit against a variety of cancers. However, it was observed that treatment with these new immune checkpoint inhibitors only worked for a subset of the cancer patients [1].

At the same time, metagenomics has elucidated the gut’s complex microbial population and provided insight on how inflammation is modulated by the different types of bacteria and its metabolites. With the link between inflammation and immunosuppression, Routy et al. [2], Gopalkrishnan et al. [3] and Matson et al. [4] independently demonstrated the contribution of the microbiome in the lack of response to immune checkpoint inhibition among these patients. Independently, they showed that positive responders to treatment had gut microbiota associated with lower inflammation while the negative responders had more bacteria associated with inflammation in the gut. These recent studies cited the role of the gut microbiome in modulating inflammation and immunosuppression which enhanced the therapeutic benefit of using immune checkpoint inhibitors such as anti-PD1 immunotherapy [2,3,4].

Inflammation is currently accepted to be central to most chronic diseases [5]. Inflammation is a double-edged sword. In its acute form, inflammation triggers the host’s immune system to confront microbial infection, repair damages and dispose cellular debris. But if inflammation persists for extended periods of time, inflammation in its chronic guise can promote the progression of several diseases such as cancer, diabetes, neurodegeneration, etc. [5]. This tenuous balance between acute and chronic inflammation is critical in keeping an individual’s state of health. In addition to the growing evidence of the role of the microbiome in regulating inflammation and the immune system, there has been increasing evidence that PUFAs have the same role. In this communication, a hypothesis on LC-PUFAs relationship with the microbiome and the immune system in modulating inflammation to control cancer, is presented.

### 1.1. Background

#### 1.1.1. Triggering and Regulating Inflammation

Long-chain polyunsaturated fatty acids such as omega (n)-3 and n-6 PUFAs are major components of cellular membranes and necessary for normal brain and neurological development. In addition to providing structural integrity to cells as major components of the cell membranes, these molecules play critical roles in cell signalling and modulating inflammation. Arachidonic acid (AA) is a major component of the cell membrane. The n-3 PUFAs, DHA (docosahexaenoic acid) and EPA (eicosapentaenoic acid), are known to be anti-inflammatory since their metabolites (protectins and resolvins) are key in resolving inflammation [6,7].

EPA and DHA are not efficiently produced by the human body from the conversion of the dietary essential fatty acid, ALA (alpha linolenic acid) and are better sourced from a diet rich in EPA and DHA. On the other hand, n-6 PUFAs especially AA is known to be inflammatory and is synthesised efficiently in humans from the dietary intake of the essential fatty acid, LA (linoleic acid) [6,7]. 

Inflammation is triggered by the enzymatic release of AA from cellular membranes upon injury leading to a metabolic cascade where pro-inflammatory eicosanoids activate the production of inflammatory cytokines that recruit immune cells to the site of injury or infection. Once immune cells arrive at the site of injury or infection, they release pro-inflammatory signals to amplify their response and complete the task. This inflammatory pathway is counter-balanced by the metabolism of n-3 fatty acids (DHA and EPA) and its metabolites to resolve the inflammatory process once the immune system has overcome the infiltrating agent or damage.

Dietary EPA and DHA, once absorbed is readily incorporated into cellular membranes. Membrane-bound EPA and DHA are released by the same phospholipase A2 enzyme that leads to the formation of anti-inflammatory eicosanoids and pro-resolving lipid mediators (resolvins and protectins), which are responsible for resolving inflammation. Furthermore, EPA directly competes with enzymes involved in AA metabolism, providing an additional handle in controlling inflammation [6,7]. Recently, endocannabinoid epoxides have been identified as omega-3 metabolites, alluding to an important role in down-regulating inflammation [8]. It has been postulated that the imbalance of inflammatory n-6 and anti-inflammatory n-3 fatty acids is responsible for most chronic diseases [9]. Moreover, the levels of AA and EPA has been used as a measure of inflammation. This may be a better indicator compared to other inflammation biomarkers because it represents the propensity for inflammation at its very early stages [10].

Anti-inflammatory drugs such as aspirin and NSAIDS (non-steroidal anti-inflammatory drugs), inhibit cyclooxygenase enzymes and prevent the synthesis of key mediators of the inflammation cascade: pro-inflammatory eicosanoids and their metabolites. Further upstream in this pathway, steroids block phospholipase activity thereby shutting both pro-inflammatory and intrinsic inflammatory pathways which explains the serious side-effects associated with steroids [6,7]. 

#### 1.1.2. Microbes and Inflammation

Historically, microbes have been viewed mainly as infectious agents that cause disease with minimal benefit to the human host. They have been one of the main triggers of inflammation and cause of disease. However, it is now recognised that there are symbiotic microbes (probiotics) that have a positive effect in human health including the modulation of inflammatory response [11]. The dynamics between beneficial and harmful microbes is now better understood. Dysbiosis relates to the imbalance of the gastrointestinal microbiome leading to the gut’s suboptimal state of health, i.e., an abnormal preponderance of harmful microbes breaching the gut barrier. This breach facilitates infiltration of these harmful bacteria and leads to conditions that sustain inflammation and chronic diseases [12]. Chronic inflammation weakens the intestinal epithelium facilitating the continuous entry of harmful bacteria, which releases factors (e.g., lipopolysaccharide, LPS) and metabolites that suppress the immune system. Studies show that particular genera of bacteria such as *Bifidobacteria* produce metabolites, especially short chain fatty acids, like butyrate, strengthen the gut barrier and regulate inflammatory response [12].

#### 1.1.3. Immune System and Inflammation

The major function of the immune system is to combat infiltrating harmful infectious agents as well as host cells, such as neoplastic cells, that have gone awry. Cytokines such as TNF-alpha, etc., are activated by the initial inflammatory response facilitating the recruitment and entry of immune cells from the lymphatic vasculature. They further react with immune cells to accelerate the inflammatory cascade by releasing other cytokines such as IL-1, IL-6, etc., as well as free radicals to complete the process of eradicating infection or repairing tissues, as well as resolving the inflammatory response. The immune cells range from members of the innate immune system (e.g., natural killer cells, macrophages, neutrophils, etc.) to members of the cellular (T-cells) and humoral (B-cells) response network as well as immune suppressor cells. However, the failure of the immune system to resolve inflammation can lead to genesis of the tumour microenvironment. An example of this shift from a pro-inflammatory anti-tumour response and shift to an immunosuppressive tumour micro environment can be gleaned from tumour-associate macrophages [13]. When low levels of inflammation persist, e.g., when LPS-containing bacteria leaks through the gut and freely infiltrate the system, immune cells continue to secrete inflammatory cytokines and a vicious cycle is created. Although inflammation may start at a low level, its prolonged presence leads to accumulated tissue and DNA damage culminating into a severe and extended state attributed to chronic diseases such as cancer, diabetes and neurological disorders. Far worse is chronic inflammation leads to immunosuppression as well as the activation of immune checkpoint pathways which promotes a tumour microenvironment [13,14].

### 1.2. Connecting the Dots

The recent discovery of the interaction between the gut microbiota and the immune checkpoint pathway [2,3,4] demonstrates a link between the gut and the immune system as well as the key role of inflammation in cancer. However, the link between microbes and cancer is not new. William Coley was the first to use bacteria as a means to stimulate the immune response to combat cancer in a large cohort of patients [15]. Furthermore, Bacillus Calmette-Guerin (BCG), a mycobacterium originally developed as a tuberculosis vaccine, has been used to treat non-invasive renal cancer via a non-specific cellular immune response. *Helicobacter pylori* was determined to be the cause of gastric cancer by Barry Marshall and Robin Warren, and is the cause of other gastric diseases [16].

These recent studies independently demonstrated how the composition of gut microbes can determine the efficacy of immune checkpoint inhibitors against cancer. This represents a novel proposition for modulating cancer and potentially other chronic diseases. It was hypothesised that dysbiosis creates an inflammatory environment in the gut and spreads systemically leading to immune suppression and that this state can be overcome by shifting the balance towards favourable microbiota thereby re-activating immune surveillance function [2,3,4]. 

These studies not only revealed a new avenue for treating cancer but also identified potential biomarkers (from the microbiome) to tailor the use of immune checkpoint inhibitors and possibly improve the efficacy of immunotherapies. Nevertheless, significant challenges remain. Multitudes of gut microbes still require identification and determination of their respective roles. Once identified, it will be necessary to propagate the desired microbial populations with the desired phenotype (s) preserved and to deliver them in a suitable, therapeutic form. 

Routy et al. [2], Gopalkrishnan et al. [3], and Matson et al. [4] independently showed that germ-free mice, which received faecal microbiome transplantation from samples of patients who responded to treatment with immune checkpoint inhibitors, also responded positively against tumour implants. However, in reality, human patients will have particular gut environments with pre-existing established microbial colonies. Colonising a recipient patient’s gut microbiota with new microbial communities will have some challenges. The new colonies will need to compete with established microbial communities and requires that they have a favourable ecological environment to survive in the new host’s gut. 

Faecal microbiome transplantation is an approach that preserves the phenotype of the desired microbiome and has shown to work against *Clostridium difficile* infections which is a single bacterial species [17]. However, many unknowns remain especially with regard to shifting the optimal balance amongst numerous microbial species as well as patient safety. Therapeutic efficacy may be achieved with a plethora of microbial permutations but determining these can be a significant undertaking. Furthermore, host genetic differences may influence which microbial populations would thrive and provide efficacy. 

Is there a simpler solution to achieve an optimal balance in the gut microbiome in order to increase the efficacy of cancer immunotherapies?

## 2. Hypothesis: n-3 and n-6 PUFAs Modulate the Microbiome and Inflammation to Activate the Immune System—Optimal n-3 and n-6 PUFA Levels Lower Inflammation and Help Achieve a Balanced Microbiome to Enhance the Immune System

**Figure 1:**
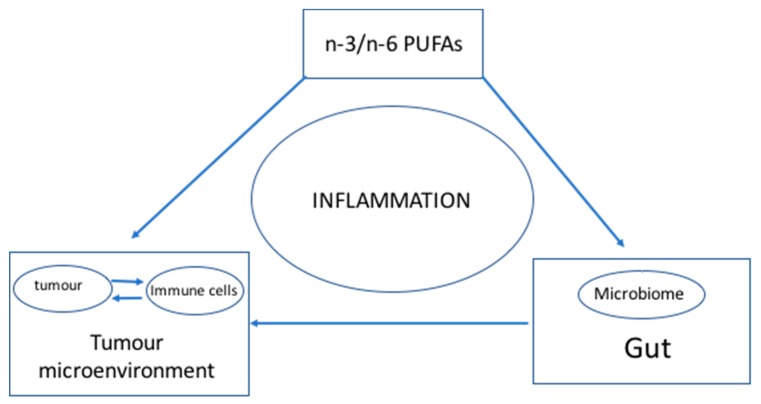
The hypothesis. Inflammation is the central element in the relationship among LC-PUFAs, the gut microbiome and the tumour microenvironment/immune system. Inflammation is regulated by the interaction of the components of the triad. LC-PUFAs (n-3 and n-6 fatty acids) have been shown to directly regulate cancer cells and immune cells as well as modulating the inflammatory state that sustains the tumour microenvironment which promotes the growth and malignancy of tumours. Simultaneously, LC-PUFAs have a direct role on microbial growth in the gut, which has consequences to the inflammatory state of the gut. Gut dysbiosis has a direct consequence in promoting inflammation in the tumour microenvironment suppressing the anti-tumour activities of the immune cells and immune checkpoint inhibitors. The model supports the role of LC-PUFAs in regulating the progression of cancer directly and indirectly through the gut microbiome assisting in modifying the tumour microenvironment.

Although the interaction between gut microbiota and the immune checkpoint pathway has been discovered [2,3,4], the mechanism of interaction needs to be elucidated. A strong, direct correlation was observed in several studies between n-3/n-6 PUFA levels and the microbiome [18], while an inverse correlation was observed for n-3 PUFA levels and inflammation [9]. Although several studies have demonstrated a direct link between n-3/n-6 levels and the microbiome and independently between n-3/n-6 levels with inflammation, the causal relationship whether n-3/n-6 levels modulate the interaction between the microbiome and the immune system has been circumstantial at best [18,19,20]. This communication hypothesises that higher n-3 PUFA and lower n-6 PUFA levels lower inflammation and promote the growth of favourable gut microbes. Consequently, a healthy gut microbiome together with optimal n-3 and n-6 PUFA levels stimulate an active immune system as well as enhances its ability to overcome immunosuppression in the tumour microenvironment. If LC-PUFA levels positively correlate with an optimal microbiome, it could be used to predict a cancer patient’s response to immunotherapy. Therefore, it is conceivable that optimal DHA and EPA as well as AA levels would promote the desired microbial population in the gut and result in a positive response to immunotherapy. The working model for the hypothesis is summarized in Figure 1.

### 2.1. Rationale behind the Hypothesis 

Elucidating direct causality for n-3 PUFAs especially DHA and EPA, provides an opportunity to confirm links with the microbiome and immunotherapy. This hypothesis can be tested because it is possible to measure n-3 PUFA levels vis-à-vis clinical endpoints. If validated, LC-PUFAs can be used as a companion diagnostic to determine whether an intervention of choice such as a particular immunotherapy would yield positive results. Hypothetically, LC-PUFA measurements could also lead to the discovery of simple fatty acid diagnostic biomarkers that would correlate with optimal microbial populations in the gut. This would be far simpler than genotyping entire microbial populations from patient stool samples. The genotyping protocol also runs the risk of missing certain microbial populations during the sampling process. 

Using n-3 and n-6 PUFAs as proxy for the microbiome has the advantage of direct intervention: the microbiome profile can be modulated prior to immunotherapy to achieve optimal efficacy. Direct intervention with n-3 PUFAs is simpler and more cost-effective compared to the use of probiotic cocktails or faecal transplantation to improve therapeutic outcomes. However, this does not preclude the possibility of combining therapeutic strategies involving n-3 PUFAs, probiotic cocktails and/or faecal transplantation. 

#### 2.1.1. n-3/n-6 PUFAs and Cancer

EPA and DHA could modulate cancer by different mechanisms: (i) apoptosis; (ii) angiogenesis and metastasis; (iii) inflammation; (iv) tumour cell proliferation; (v) epigenetic abnormalities; and (vi) gene expression and cell signalling [18,19,20]. In addition, an anti-neoplastic mechanism was recently discovered in endocannabinoid epoxides, n-3 metabolites which have tumour cell-killing capabilities as well anti-angiogenic properties that slow down tumour growth and migration [21]. Although most of the aforementioned mechanisms were observed from in vitro or animal models, there is increasing clinical evidence showing that adjunct intervention with n-3 fatty acids leads to better recovery among patients after chemotherapy and surgery [18,22]. It was also recently proposed that n-3 PUFAs can modulate cancer in combination with immunotherapy based on the activation of tumour-killing cytokines in an inflammatory environment as well as overcoming immunosuppression simultaneously [18,23]. Recent studies have shown that low levels of the n-3 fatty acids (EPA and DHA) and high n-6 PUFAs, especially AA, are associated with increased incidence of having cancer [24]. The proposed role of LC-PUFAs in regulating inflammation and the gut microbiome may be critical in assisting the immune system in recognising tumour microenvironments possibly even preventing metastasis.

It should also be noted that n-3 PUFAs’ with its strong anti-inflammatory properties should have a window of optimal use before it becomes a double-edged sword with potentially serious adverse events. Based on in vitro and animal models as well as limited clinical data, there are concerns that excess n-3 PUFAs may promote cancer. More clinical trials where precise measurements of n-3 PUFA levels can be taken can hopefully define the optimal window and shed light on the mechanisms on how they affect the microbiome [19]. 

#### 2.1.2. n-3 PUFAs and the Microbiome

A direct link has been established between n-3 PUFAs and modulation of the microbiome especially with respect to inflammation [12,18,25]. Firstly, EPA has been shown to have anti-microbial properties with cytotoxic effects against several bacterial species such as *Propionibacterium acnes*, *Staphylococcus aureus*, and *Bacillus cereus* [26]. EPA and DHA has shown to have multiple mechanisms in regulating *H. pylori* infection with direct implications to gastric cancer [16].

There has also been significant progress in understanding mechanism of how LC-PUFAs regulate the gut’s microbial population. When mice were fed pro-inflammatory n-6 PUFA-enriched diets, it induced metabolic endotoxemia, which is a result of gut dysbiois [26]. Studies were also conducted on FAT-1 mice, a transgenic rodent that overexpresses an enzyme derived from the worm *Caenorhabditis elegans* that converts n-6 to n-3 PUFA constitutively without the need for dietary n-3 supplementation. FAT-1 mice fed with an n-6 PUFA enriched diet had reduced low grade inflammation and metabolic endotoxemia. It was observed that levels of intestinal alkaline phosphatase (IAP) was enhanced throughout the animals’ tissues [27]. Increased IAP activity has been shown to be responsible for modifying gut microbiota resulting in reduced LPS production and gut permeability which leads to relief from metabolic endotoxemia and inflammation [28]. IAP removes phosphates from molecules, such as LPS, so that when they infiltrate undesirable microbes, the toxic and inflammatory properties of LPS are inactivated. This is consistent with a recent study where orally-administered IAP modified the microbiome and led to the improvement of the chronic inflammatory condition caused by *Salmonella* infection [28]. Moreover, studies have shown an inverse relationship between inflammation and IAP activity in stools of diabetic patients [29]. n-3 PUFA may be an upstream regulator of IAP expression and if so, would be one probable mechanism of how n-3 PUFAs regulate inflammation through the gut microbiota. 

Transgenic zebrafish expressing salmon desaturase genes fish had enhanced levels of n-3 PUFAs, resulting to an increase in anti-bacterial and anti-inflammatory activities [30]. Moreover, there is evidence that n-3 fatty acids directly modulate inflammasome activation in macrophages to prevent inflammation [31], while the inflammasome plays a role in regulating the microbial composition in the gut [32]. Thus, n-3 PUFAs may modulate the microbiome by other mechanisms in addition to upregulation of IAP but it is possible that these are all interconnected.

These mechanistic studies are supported by recent observational studies in humans. In middle aged and elderly women, n-3 fatty acid levels correlated with gut microbiome diversity, independent of fibre intake and the production of *N*-carbamylglutamate, a metabolite known to have beneficial effects to the mammalian gut [33]. Furthermore, from a case report, the gut microbiota of a healthy, physically active 45-year old male showed changes after taking 600 mg of n-3 PUFAs daily for 14 days [34]. There was a decrease in microbial diversity but an increase in species that produce butyrate, a short chain fatty acid known to promote gut health. Interestingly, the microbiome reverted to its original state after a 14-day washout of n-3 PUFAs [34]. A randomised, cross-over trial on healthy human subjects treated with different n-3 PUFA formulations did not lead to major shift in microbial diversity but an increase in butyrate producing bacteria was observed [25].

#### 2.1.3. n-3 PUFAs and the Immune System

Omega-3 PUFAs are known to be anti-inflammatory and able to suppress the immune system. Thus, it has been suggested that n-3 PUFAs should be contraindicated in conditions that require an active immune system such as during an infection and even in cancer since further immunosuppression would worsen the prognosis in cancer therapy [20]. However, as pointed out earlier, inflammation has a role in promoting immunosuppression in the tumour microenvironment. Furthermore, recent studies indicate that even though n-3 PUFAs may be anti-inflammatory, it has immune-stimulating effects in both the humoral and cellular immune systems [35,36]. There is mounting evidence on how n-3 PUFAs can inhibit tumour growth and progression [18,19,20]. These observations contradict preconceived fears in using n-3 PUFAs to treat cancer. The current body of evidence suggests that n-3 PUFAs perform a balancing act where it has both pro-inflammatory functions (overcome immunosuppression in the tumour microenvironment), as well as anti-inflammatory functions (modulate neoplastic progression).

### 2.2. Testing the Hypothesis

#### 2.2.1. Measuring the Levels of PUFAs and IAP: Omega-3 Index, AA/EPA Ratio and IAP

The ability to measure levels of the LC-PUFAs especially EPA, DHA and AA in the body is key to testing the hypothesis as well as the applying it in clinical settings. 

Once absorbed from dietary intake, LC-PUFAs are readily incorporated into cellular membranes. LC-PUFAs are present in plasma as free fatty acid and phospholipids only in a matter of minutes to days, which makes sampling difficult. The other extreme is that in adipose tissues the EPA/DHA ratio remains stable for months, even years but sampling can be very challenging. LC-PUFAs however are present in red blood cells (RBCs) at a steady-state from weeks to months and its membrane composition are a good representative of all tissue types in the body [37]. Both these factors make measurement of LC-PUFAs from blood samples the most practical. It is also convenient that blood sampling of metabolites as a proxy for disease is a common diagnostic protocol.

Measuring fatty acids in red blood cells traditionally involved the isolation of red blood cells from whole blood and the relative amounts of fatty acids determined by gas chromatography. Whole blood had to be stored at −190 °C to prevent EPA and DHA decay. This method is tedious which has limited its application in clinical trials. Currently, dried blood spot cards can be used to omega-3 levels from a few drops of blood; the EPA and DHA are stabilised at ambient temperature for up to 9 weeks with low background levels [38,39]. The LC-PUFA measurements taken by this method were comparable with those made from whole blood and red blood cell membranes [39].

The omega-3 index which corresponds to the percentage of EPA and DHA in RBC membranes was formally developed and used as a biomarker for cardiovascular health [40,41]. Although an omega-3 index of >8% has been set as a target for good cardiovascular health, it has been observed that an omega-3 index >10% is associated with better insulin sensitivity among overweight males [42]. This suggests that the omega-3 index as a biomarker may vary for different metabolic states and diseases, such as cancer.

Another biomarker associated with the relative fatty acid content in blood is the AA/EPA ratio. Since the two PUFAs are key membrane components that regulate inflammatory response, their relative amounts are used as an inflammation marker where some studies have shown strong links in diseases such as cancer [24]. As previously described, IAP is a promising biomarker for the state of the gut microbiome and inflammation; it has been shown to directly modulate dysbiosis. It can be measured by a simple colorimetric assay from stool samples [29]. If it can be demonstrated in a controlled clinical trial that increased n-3 fatty acid intake can elevate IAP activity, omega-3 supplementation would be more cost-effective compared to using IAP which is more expensive [28,29]. 

#### 2.2.2. The Need to Measure Baseline and Endpoints

The debate on whether omega-3 supplements work continue to rage in spite of numerous clinical trials and meta-analyses [43,44]. Most of the clinical trials conducted with omega-3 supplements lack baseline and end of treatment measurements [44,45]. The lack of measurements can be attributed to the tedious nature of the traditional method for fatty acid analysis of RBCs. With the availability of a cost-effective and straightforward solution in sampling with dried blood spot cards, baseline and clinical endpoints can be measured and this debate will hopefully be resolved. It should also facilitate the testing of the presented hypothesis in a number of clinical settings.

#### 2.2.3. Validating the Hypothesis

Validating the hypothesis that optimal n-3 and n-6 PUFAs would improve cancer immunotherapy outcomes would require the determination of baseline omega-3 indices and the AA/EPA ratios of cancer patients as preliminary baseline data. Cohorts of cancer patients within specified omega-3 indices and AA/EPA ratios will be determined, have their microbiomes genotyped and IAP activity measured to establish any correlations. Subsequently, the subjects will be treated with immunotherapy (e.g., immune-checkpoint inhibitors) and their responses determined. It is hypothesised that patients with high omega-3 indices and low AA/EPA ratios will correlate with increased IAP activity, i.e., favourable microbiome profiles, and respond better to immunotherapy. These results would represent an observational study that would support the hypothesis. Nevertheless, these studies should provide the information needed to define the optimal omega-3 index and AA/EPA ratio that results in a favourable microbiome that makes immunotherapy work. 

To validate the hypothesis that LC-PUFAs are key modulators of the inflammation triad, it is important to demonstrate causality. Randomised double-blind, placebo-controlled clinical trials involving cancer patients with suboptimal omega-3 indices and AA/EPA ratios with corresponding microbiome profiles, need to be conducted. Although n-3 PUFA treatment of healthy individuals did not lead to major shift in microbial diversity [25], this is consistent with response of healthy subjects with optimal metabolic parameters to treatment with resveratrol [46], while subjects with suboptimal metabolic parameters are more responsive to treatment with a polyphenol rich fraction from filtered molasses concentrate [47]. It is postulated that subjects with dysbiosis will have a stronger response to n-3 PUFA treatment leading to major shifts in microbial diversity. Therefore, patients pre-treated with n-3 PUFAs to reach the optimal omega-3 index and AA/EPA ratio followed by immunotherapy will be expected to have a higher positive response rate to immunotherapy as well as increased IAP activity and an improved microbiome profile compared to subjects who did not receive n-3 PUFAs (placebo). Moreover, it should be noted that any positive effects from n-3 PUFA treatment may also include contributions from collateral benefits due to n-3 PUFAs’ other anti-neoplastic properties [18,19,20].

Results from these trials may have implications in developing appropriate cancer treatment protocols as well as in making recommendations for the target omega-3 index and AA/EPA ratio for cancer prevention. It would also be of interest to consider healthy subjects who have inherited genetic predispositions to cancer but with no manifestation yet, and determine whether maintaining optimal levels of n-3 and n-6 PUFAs, healthy microbiome and balanced inflammation can prevent them from developing cancer. This represents a novel example of precision medicine and nutrition.

## 3. Conclusions

This communication puts forward the hypothesis that LC-PUFAs can act as proxies for determining the state of the gut microbiota. In particular, LC-PUFAs can serve as biomarkers for a cancer patient’s gut microbiome which can be modulated accordingly to improve immunotherapy outcomes. Validation of the hypothesis has implications towards disease prevention. Once validated, in order to prevent disease, cancer in this example, an individual would need to maintain the target level of LC-PUFAs in tissues as well as the level of inflammation required to maintain healthy gut microbiota and immune system. The proposed measurement of LC-PUFAs provides a practical tool to monitor and modulate the microbiome towards increased immunotherapy efficacy. LC-PUFAs and inflammation levels via the AA/EPA ratio are quantifiable parameters and should facilitate achieving and maintaining personal target levels through cognitive behavioural or dietary modification, n-3 PUFA supplementation or therapeutic intervention. One caveat is that achieving optimal n-3 and n-6 levels may take time (months) and should be a consideration in future clinical trial designs. 

Although the hypothesis has focused on an application in cancer, it is conceivable that the same principles would apply to other inflammatory disorders which have shown similar correlation between LC-PUFA levels and the microbiome: Inflammatory bowel disease [28,48], post-traumatic stress disorder [49,50], depression [51,52], non-alcoholic steatohepatitis (NASH) [32,53], and diabetes [29,54]. Similar strategies as described above can be applied to these disease indications.
